# The cross-race effect in automatic facial expression recognition violates measurement invariance

**DOI:** 10.3389/fpsyg.2023.1201145

**Published:** 2023-12-07

**Authors:** Yen-Ting Li, Su-Ling Yeh, Tsung-Ren Huang

**Affiliations:** ^1^Department of Psychology, National Taiwan University, Taipei City, Taiwan; ^2^Graduate Institute of Brain and Mind Sciences, National Taiwan University, Taipei City, Taiwan; ^3^Neurobiology and Cognitive Science Center, National Taiwan University, Taipei City, Taiwan; ^4^Center for Artificial Intelligence and Advanced Robotics, National Taiwan University, Taipei City, Taiwan

**Keywords:** cross-cultural psychology, cross-race effect, emotion recognition, facial expression, inductive bias, measurement invariance, validation

## Abstract

Emotion has been a subject undergoing intensive research in psychology and cognitive neuroscience over several decades. Recently, more and more studies of emotion have adopted automatic rather than manual methods of facial emotion recognition to analyze images or videos of human faces. Compared to manual methods, these computer-vision-based, automatic methods can help objectively and rapidly analyze a large amount of data. These automatic methods have also been validated and believed to be accurate in their judgments. However, these automatic methods often rely on statistical learning models (e.g., deep neural networks), which are intrinsically inductive and thus suffer from problems of induction. Specifically, the models that were trained primarily on Western faces may not generalize well to accurately judge Eastern faces, which can then jeopardize the measurement invariance of emotions in cross-cultural studies. To demonstrate such a possibility, the present study carries out a cross-racial validation of two popular facial emotion recognition systems—FaceReader and DeepFace—using two Western and two Eastern face datasets. Although both systems could achieve overall high accuracies in the judgments of emotion category on the Western datasets, they performed relatively poorly on the Eastern datasets, especially in recognition of negative emotions. While these results caution the use of these automatic methods of emotion recognition on non-Western faces, the results also suggest that the measurements of happiness outputted by these automatic methods are accurate and invariant across races and hence can still be utilized for cross-cultural studies of positive psychology.

## 1 Introduction

Facial emotional expressions are social signals in nature and essential for successful social interactions. People tend to have more or intensified facial emotional expressions in the presence of others (Blair, 2003; Frith, 2009). Subsequently, the perceivers of these expressions can then infer the expressers’ mental states to determine whether to approach or avoid the emotional expressers (Seidel et al., 2010; Lowe and Ziemke, 2011). People with limited facial emotion expressions, such as those with facial paralysis, are often perceived negatively and hence have trouble building interpersonal relationships (Blair, 2003; Trevisan et al., 2018). Similarly, social robots that lack facial emotional expressions are difficult for humans to interact with (Pessoa, 2017; Stock-Homburg, 2022).

Facial emotion expressions and their recognition have been extensively studied in psychology and other emotion-related fields. The most influential series of studies on this topic comes from Ekman (1970, 1971), Ekman and Friesen (1986), and Ekman and Cordaro (2011) who proposed the existence of six to seven basic emotions—anger, surprise, disgust, happiness, fear, sadness, and contempt. Moreover, each of these basic emotions has a corresponding facial expression, which is universally produced and recognized across different cultures (Ekman et al., 1987; Izard, 1994; Wang et al., 2006; Matsumoto et al., 2009). Although there have been recent studies challenging the universal emotion-expression links (Barrett et al., 2019; Durán and Fernández-Dols, 2021; Tcherkassof and Dupré, 2021), such basic emotion view still serves as the theoretical basis in many applied studies of emotion.

Facial emotional expressions can be analyzed in terms of movements of facial muscles. The well-known Facial Action Coding System (FACS) defines action units (AUs) as the fundamental actions of individual muscles or groups of muscles (Rosenberg and Ekman, 2020). In FACS, the expression of each basic emotion can then be characterized by the joint movements of a specific set of AUs. Because FACS is an anatomically based, relatively objective coding system of facial emotion expressions, it is a popular scheme for coding facial expressions in research or applications. Note, however, that it requires intensive training to master FACS and intensive labor to apply FACS. Moreover, human coders may be inconsistent in judging the movements of AUs.

**FIGURE 1:**
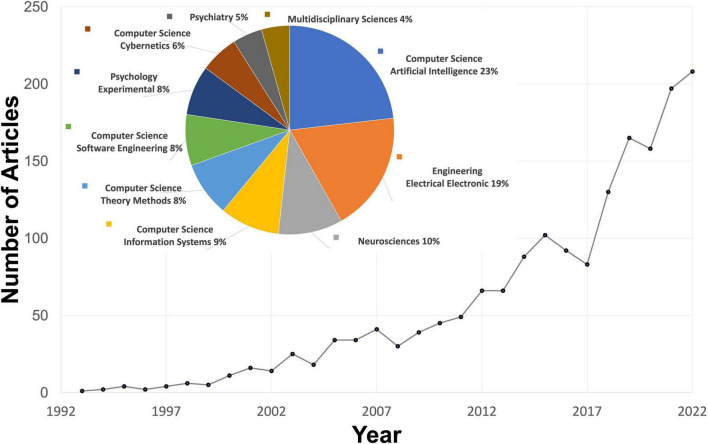
The number of articles mentioning automatic facial expression recognition. The results were obtained from web of science using the search: (automated OR automatic) AND (coding OR recognition OR analysis) AND facial AND (emotions OR expressions OR emotional expressions).

To overcome the issues of time consumption and subjectivity in the manual coding of facial expressions, computer vision techniques for automatic facial expression recognition have been actively developed by computer scientists and engineers and gradually adopted by behavioral scientists (Figure 1). For example, automatic facial expression recognition systems (AFERSs) have been applied as a clinical tool to assess the abilities of emotional reactivity, regulation, or expression in people (Flynn et al., 2020), such as those with autism spectrum disorder (Owada et al., 2018; Manfredonia et al., 2019), personality disorder (De Meulemeester et al., 2022), and social anxiety disorder (Pearlstein et al., 2019). Overall, these AFERSs facilitate large-scale assessments of facial expressions in real-time cameras or recorded images/videos.

Automatic facial expression recognition systems are powered by various computer vision techniques. A traditional machine learning-based AFERS often consists of three processing phases—image preprocessing (e.g., cropping a face out of a background), feature extraction (e.g., extracting geometry-based features), and expression classification (e.g., using a support vector classifier). By contrast, recent AFERSs based on deep-learning (DL) neural networks are end-to-end systems that automatically learn to extract facial features for accurate expression classification and hence reduce the need for image preprocessing and feature extraction (O’Mahony et al., 2020; Khan, 2022). While DL-based AFERSs require a large number of face images as training data, they outperform traditional approaches and have become state-of-the-art (Li and Deng, 2020; Ekundayo and Viriri, 2021).

Automatic facial expression recognition systems that leverage machine learning, be they traditional or DL-based, all rely on inductive learning of statistical regularities in images. Due to this inductive nature, such AFERSs often suffer from problems of induction and exhibit inductive biases (Huang, 2019). For example, because these AFERSs have fewer chances to see and learn the statistical regularities of minority groups during model training, they tend to make erroneous predictions for gender, age, or racial groups that are underrepresented in the training data (Huang et al., 2023). Therefore, it is possible that popular AFERSs developed in the West are trained mostly on Western faces and less versed in Eastern faces. In consequence, these AFERSs may frequently misrecognize emotional expressions on Asian faces. This can then jeopardize the validity of clinical assessments and emotion studies that draw conclusions from automatic FER of Eastern faces.

Because of the research tendency toward measuring the behavior of non-Western people in natural rather than laboratory settings (Henrich et al., 2010; Box-Steffensmeier et al., 2022), it is anticipated that AFERSs will be increasingly used to help assess spontaneous emotional expressions in the wild for not only Western but also Eastern people. From a methodological point of view, it is then important to check whether these AFERSs evaluate the same emotional expression differently for different groups of people, as such a violation of measurement invariance will preclude meaningful interpretations of research results, especially for cultural or cross-cultural studies. For this reason, the present research sets out to examine whether AFERSs are comparably accurate in recognizing emotional expressions on Western vs. Eastern faces, as detailed below.

## 2 Materials and methods

### 2.1 Face datasets

**FIGURE 2:**
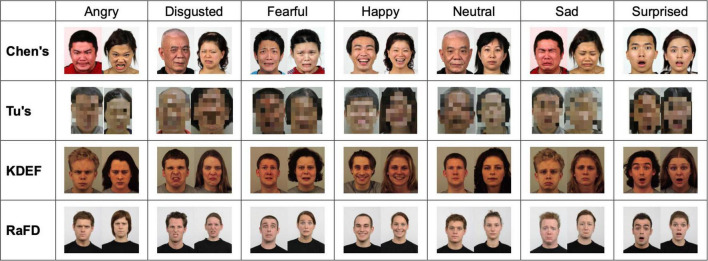
Emotional faces sampled from the four datasets. Chen’s dataset is in the public domain and can be obtained upon request from the owner, Prof. Chien-Chung Chen (Chen et al., 2013). Tu’s dataset has been reproduced here with permission and can be obtained upon request from the owner, Prof. Joshua O. Goh (Tu et al., 2018). These images have been blurred at the request of Prof. Goh. KDEF images are available in the public domain for use in scientific publications (Lundqvist et al., 1998, available at: https://kdef.se/). RaFD images are available to researchers upon request and reproduction in scientific publications is permitted (Langner et al., 2010, available at: https://rafd.socsci.ru.nl/RaFD2/RaFD?p=main).

We used four publicly available face image datasets (two Western and two Eastern) to evaluate two popular AFERSs (one commercial and one open-source). These datasets were chosen for comparison because they were constructed under similar conditions—these face photos were all taken from specific angles of human models who were asked to pose researcher-designated basic emotions against a clean background in a well-lit room. Compared to facial emotional expressions captured by cameras in the wild, these face photos shot in laboratory-controlled conditions are higher in overall quality and reduce the confounding of photographing conditions in our Western vs. Eastern comparisons. Examples of these face images are shown in Figure 2.

#### 2.1.1 Western faces

##### 2.1.1.1 RaFD

The Radboud Faces Database (RaFD) is an image dataset containing 8,040 images of 67 human models (Langner et al., 2010). Each model expressed eight emotions (Ekman’s seven basic emotions plus neutral) with three gaze directions. Each emotional expression was shot from five camera angles. These models were male and female Caucasian adults and children, as well as male Moroccan Dutch adults. The children and male Moroccan Dutch adult images were excluded from further analyses.

##### 2.1.1.2 KDEF

The Karolinska Directed Emotional Faces (KDEF) is an image dataset containing 4,900 images of human 70 human models (Lundqvist et al., 1998). Each model expressed seven emotions (Ekman’s six basic emotions plus neutral), and the emotion category label of each image was validated by a separate study (Goeleven et al., 2008). These models were 35 Caucasian males and 35 Caucasian females aged between 20 and 30 years old. Each emotion of each human model was shot from five camera angles.

#### 2.1.2 East Asian faces

##### 2.1.2.1 Chen’s data

This dataset contains 2,273 images from 29 professional performers (Chen et al., 2013). Each performer expressed seven emotions (Ekman’s six basic emotions plus neutral). These images were taken from three different viewing angles. The models were 14 Taiwanese males and 15 Taiwanese females aged between 19 to 67 years old.

##### 2.1.2.2 Tu’s data

This dataset contains 426 images from 61 human models (Tu et al., 2018). Each model expressed seven emotions (Ekman’s six basic emotions plus neutral). All the images in this dataset were taken from a straight viewing angle. The models were 20 young Taiwanese adults (10 males and 10 females) and 41 older Taiwanese adults (20 males and 21 females).

### 2.2 AFERSs

There are commercial and open-source options for AFERSs. Commercial options include FaceReader (Noldus), Affdex (Affectiva), and Facet (iMotions). While FaceReader is not the most accurate one among these commercial options (Dupré et al., 2020), it is the most popular and hence impactful one in the literature, as evidenced by the number of related articles retrieved by Google Scholar (Noldus FaceReader: 1,780; Affectiva Affdex: 738; iMotions Facet: 763 as of 2/4/2023). Notable open-source options include DeepFace, Py-Feat, and EmoPy. While DeepFace is not the most accurate one among these open-source options (Sampaio et al., 2022), it is the most popular one in the literature, as suggested by the number of related articles retrieved by Google Scholar (DeepFace: 7,800; EmoPy: 81; Py-Feat: 58 as of 2/4/2023). Therefore, we chose FaceReader v9 and DeepFace v0.0.79 as the AFERSs to be evaluated.

#### 2.2.1 FaceReader

FaceReader is a user-friendly and versatile software for analyzing face images or videos. It uses deep-learning models to detect faces and classify facial expressions. It also leverages computer vision algorithms to automate FACS scoring for estimating the emotional valence and arousal of a facial expression. Its emotion classification and FACS scoring have been shown to be valid (Lewinski, 2015; Skiendziel et al., 2019). Note that there are multiple face models available in FaceReader, including a general model for most circumstances and models for East Asian people, the elderly, and children. In the present study, we evaluated FaceReader by using the general model on the two Western datasets and the East Asian model on the two East Asian datasets.

#### 2.2.2 DeepFace

DeepFace is a lightweight Python package that implements face recognition and facial attribute analysis (age, gender, emotion, and race). It is a hybrid framework integrating several state-of-the-art models based on convolutional neural networks, such as VGG-Face, FaceNet, OpenFace, DeepID, ArcFace, Dlib, and SFace. For FER, the face in an inputted image will be first located by a face detection model, and the emotional expression of the detected face will then be categorized by an emotion recognition model. Specifically, we used DeepFace version 0.0.79 with the best-performing RetinaFace as the face detector and the built-in convolutional neural network as the emotional recognizer, which had been pre-trained on the FER-2013 dataset (Serengil and Ozpinar, 2021).

### 2.3 Validation procedure

We validated FaceReader’s and DeepFace’s judgments on the emotion categories of facial expressions. The emotion category label of each image in all the datasets had been validated by human raters in earlier studies and served as the ground truth in the present study. We could then calculate the classification accuracy for each AFERS on face images from the same emotion category of each dataset. Here, we primarily used frontal-view faces with a straight gaze direction to estimate the upper-bound performances of these expression classifications. Nonetheless, we also tested the AFERSs on frontal-view faces with averted gazes for comparison, as direct gaze might bias perception toward approach-oriented emotions (anger and happiness), and averted eye gaze might bias perception toward avoidance-oriented emotions (scare and sadness) (Adams and Kleck, 2005).

To control for image quality in the comparison across datasets, we only analyzed images that were most unequivocally recognized by humans. Specifically, we only kept an image with a rate of at least 90% human agreement on the dominant emotion category of the face image. For the two Western datasets, the human agreement rate of each image was calculated from emotion classification results (KDEF: 272 raters; RaFD: 276 raters) and made available by previous validation studies (Goeleven et al., 2008; Langner et al., 2010). For the two Eastern datasets, the human agreement rate of each image wasn’t provided by the previous validation studies where human raters were asked to estimate the intensity of each emotion category rather than to recognize the dominant emotion category for each face image. Nonetheless, the dominant emotion category of each face image for each human rater could be defined as the emotion with the highest intensity rating across categories. Accordingly, we could still calculate the human agreement rates for all but the neutral face images in the two Eastern datasets (Chen’s: 400 raters; Tu’s: 4 mean raters representing the four sex-by-age groups) as these neutral images had no intensity ratings.

## 3 Results

AFERSs return null results for images they cannot process. This only happened to DeepFace, which produced null results for 3 images (5.9%) of Chen’s dataset. These images were excluded from further analyses and not counted as incorrect cases.

The overall accuracy of each AFERS on images with a 100% human agreement rate is shown in Table 1. For each AFERS, proportion *z*-tests were conducted to compare the classification accuracies between East Asian datasets and Caucasian datasets. For both AFERSs, the classification accuracy of Caucasian datasets is significantly greater than that of East Asian datasets [Chi-Square tests on the numbers of correctly vs. incorrectly recognized expressions as a function of *race*: FaceReader: χ^2^ (1, *N* = 262) = 13.22, *p* < 0.001, ϕ = 0.22; DeepFace: χ^2^ (1, *N* = 259) = 7.29, *p* = 0.007, ϕ = 0.17].

**TABLE 1:** The mean classification accuracy of each AFERS on images with a 100% agreement rate.

Group	N_images_	FaceReader	N_images_	DeepFace
East Asian	173	0.85	170	0.71
Caucasian	89	1	89	0.87

We further investigated how well the two AFERSs could recognize different emotions. In these later investigations, the image quality criterion was relaxed from 100% to 90% human agreement rate to increase the sample size for statistical inferences. In spite of this adjustment, only few disgusted and fearful face images were available for further analyses (disgusted: Chen:7, Tu:1, KDEF:24, RaFD:3; fearful: Chen:0, Tu:1, KDEF:0, RaFD:17). As a result, we only analyzed images of happy, sad, angry, and surprised faces hereafter. On this new set of images, DeepFace produced null results for 9 images (2.5%) of Chen’s dataset, including the 3 images already excluded in Table 1.

The by-emotion classification results are summarized in Table 2. Overall, FaceReader significantly outperformed DeepFace regardless of the emotion categories and datasets [Chi-Square test on the numbers of correctly vs. incorrectly recognized expressions as a function of *AFERS*: χ^2^ (1, *N* = 1,403) = 45.25, *p* < 0.001, ϕ = 0.18], with the classification accuracies being 92.6% and 80.2%, respectively. On the Western datasets, FaceReader was overall 99.6% correct, while DeepFace was only 87.1% correct. On the Eastern datasets, the overall classification accuracies of FaceReader and DeepFace dropped significantly to 89% and 76.5% correct, respectively [Chi-Square tests on the numbers of correctly vs. incorrectly recognized expressions as a function of *race*: FaceReader: χ^2^ (1, *N* = 706) = 24.38, *p* < 0.001, ϕ = 0.19; DeepFace: χ^2^ (1, *N* = 697) = 10.50, *p* = 0.001, ϕ = 0.12]. Taken together, these results show that both AFERSs provided considerably different measurements of the same facial expressions for different racial groups, violating measurement invariance. A close examination of the results reveals that regardless of the datasets, happy and surprised expressions were significantly easier to recognize than negative expressions (i.e., sadness and anger) for both the AFERSs [Chi-Square tests on the numbers of correctly vs. incorrectly recognized expressions as a function of *emotional valence*: FaceReader: χ^2^ (1, *N* = 706) = 36.80, *p* < 0.001, ϕ = 0.23; DeepFace: χ^2^ (1, *N* = 697) = 15.44, *p* < 0.001, ϕ = 0.15]. Specifically, FaceReader and Deepface were, respectively, 96.2% and 83.8% correct about non-negative expressions but only 82.1% and 69.8% correct about negative expressions.

**TABLE 2:** The mean classification accuracy of each AFERS on faces of the same expression.

Expression	Dataset	FaceReader			DeepFace		
		N_images_	Accuracy		N_images_	Accuracy	
Angry	Chen’s	19	0.895	0.647	19	0.737	0.618
	Tu’s	15	0.333		15	0.467	
	KDEF	31	0.968	0.982	31	0.903	0.857
	RaFD	25	1.000		25	0.800	
Happy	Chen’s	214	0.967	0.955	207	0.923	0.885
	Tu’s	54	0.907		54	0.741	
	KDEF	59	1.000	1.000	59	1.000	1.000
	RaFD	39	1.000		39	1.000	
Sad	Chen’s	34	0.706	0.587	34	0.441	0.478
	Tu’s	12	0.250		12	0.583	
	KDEF	20	1.000	1.000	20	0.800	0.791
	RaFD	23	1.000		23	0.783	
Surprised	Chen’s	78	0.923	0.932	76	0.724	0.652
	Tu’s	39	0.949		39	0.513	
	KDEF	17	1.000	1.000	17	0.882	0.682
	RaFD	27	1.000		27	0.556	
Total	Chen	345	0.928	0.890	336	0.818	0.765
	Tu	120	0.783		120	0.617	
	KDEF	127	0.992	0.996	127	0.929	0.871
	RaFD	114	1.000		114	0.807	

The mean accuracies on the two Eastern datasets (i.e., Chen’s and Tu’s) and on the two Western datasets (i.e., KDEF and RaFD) are also presented for comparison. With the agreement criterion of 0.9, DeepFace returned null results for 7 happy faces and 2 surprised faces in Chen’s dataset.

We also inspected whether the measurement invariance was violated across not only race but also sex and age. The results of different sex groups are summarized in Table 3. Overall, both FaceReader and DeepFace classified facial expressions equally accurately for male and female faces, regardless of the datasets used [Chi-Square tests on the numbers of correctly vs. incorrectly recognized expressions as a function of *sex*: FaceReader: χ^2^ (1, *N* = 706) = 0.012, *p* = 0.91, ϕ = 0.004; DeepFace: χ^2^ (1, *N* = 697) = 0.007, *p* = 0.93, ϕ = 0.003]. As for age, the two Eastern but not the two Western datasets contain faces of older adults, and the mean classification accuracies of different age groups averaged across the two Eastern datasets are summarized in Table 4. Overall, both FaceReader and DeepFace classified facial expressions more accurately for young than older faces [Chi-Square tests on the numbers of correctly vs. incorrectly recognized expressions as a function of *age group*: FaceReader: χ^2^ (1, *N* = 465) = 13.92, *p* < 0.001, ϕ = 0.17; DeepFace: χ^2^ (1, *N* = 456) = 12.44, *p* < 0.001, ϕ = 0.17]. Specifically, FaceReader and DeepFace were, respectively, 91.8% and 79.9% correct about young faces but only 77.3% and 61.0% correct about older faces.

**TABLE 3:** The mean classification accuracy of each AFERS on faces of the same sex and expression.

Expression	Dataset	FaceReader				DeepFace			
		N_images_	Male	N_images_	Female	N_images_	Male	N_images_	Female
Angry	Chen’s	12	0.833	7	1.000	12	0.833	7	0.571
	Tu’s	8	0.250	7	0.429	8	0.500	7	0.429
	KDEF	19	1.000	12	0.917	19	0.842	12	1.000
	RaFD	14	1.000	11	1.000	14	0.857	11	0.727
Happy	Chen’s	106	0.962	108	0.972	100	0.870	107	0.972
	Tu’s	27	0.926	27	0.889	27	0.778	27	0.704
	KDEF	28	1.000	31	1.000	28	1.000	31	1.000
	RaFD	20	1.000	19	1.000	20	1.000	19	1.000
Sad	Chen’s	11	0.455	23	0.826	11	0.273	23	0.522
	Tu’s	2	0.000	10	0.300	2	0.500	10	0.600
	KDEF	9	1.000	11	1.000	9	0.778	11	0.818
	RaFD	10	1.000	13	1.000	10	0.800	13	0.769
Surprised	Chen’s	35	0.914	43	0.930	33	0.758	43	0.698
	Tu’s	21	0.952	18	0.944	21	0.571	18	0.444
	KDEF	5	1.000	12	1.000	5	1.000	12	0.833
	RaFD	14	1.000	13	1.000	14	0.643	13	0.462
Total	Chen’s	164	0.909	181	0.945	156	0.801	180	0.833
	Tu’s	58	0.810	62	0.758	58	0.655	62	0.581
	KDEF	61	1.000	66	0.985	61	0.918	66	0.939
	RaFD	58	1.000	56	1.000	58	0.845	56	0.768

**TABLE 4:** The mean accuracies of each AFERS on faces of the same age group in Chen’s and Tu’s datasets.

Expression	FaceReader				DeepFace			
	N_images_	Older	N_images_	Young	N_images_	Older	N_images_	Young
Angry	12	0.250	22	0.864	12	0.333	22	0.773
Happy	45	0.911	223	0.964	39	0.846	222	0.892
Sad	7	0.286	39	0.641	7	0.571	39	0.462
Surprised	24	0.917	93	0.935	24	0.375	91	0.725
Total	88	0.773	377	0.918	82	0.610	374	0.799

Finally, we also examined whether gaze direction affected the classification accuracy of the two AFERSs using RaFD, the only dataset in the present study that provides faces with different gaze directions. Table 5 summarizes the results of varying gaze directions for each emotion category. Overall, both FaceReader and DeepFace recognized facial expressions equally accurately for faces with direct or averted gazes [Fisher’s exact tests on the numbers of correctly vs. incorrectly recognized expressions as a function of *gaze direction*: FaceReader: Angry: *p* = 1.00; Happy: *p* = 1.00; Sad: *p* = 1.00; Scared: *p* = 0.001; DeepFace: Angry: *p* = 0.74; Happy: *p* = 1.00; Sad: *p* = 0.74; Scared: *p* = 0.77]. In other words, gaze direction did not bias machine perception toward either approach-oriented emotions (anger and happiness) or avoidance-oriented emotions (scare and sadness).

**TABLE 5:** The mean accuracies of each AFERS on faces of the same gaze direction in RaFD.

Expression	FaceReader				DeepFace			
	N_images_	Direct	N_images_	Averted	N_images_	Direct	N_images_	Averted
Angry	25	1.000	37	1.000	25	0.800	37	0.838
Fearful	18	0.944	36	1.000	17	0.529	36	0.611
Happy	39	1.000	76	1.000	39	1.000	76	1.000
Sad	23	1.000	36	1.000	23	0.783	36	0.833
Total	95	0.989	185	1.000	104	0.827	185	0.859

## 4 Discussion

The present study found that both commercial and open-source AFERSs developed in the West were more accurate in recognizing emotional expressions on Western than Eastern faces. The lower accuracy of these AFERSs on Eastern faces, especially those of older adults, can compromise the validity of clinical assessments and studies that utilize these systems to analyze Eastern populations, such as those addressing cultural influences or cross-cultural differences in emotion recognition. Therefore, this violation of measurement is a threat worth noticing and further investigating.

The discrepancy in the recognition accuracy across races may not be attributed to the differences in the facial bone structure across races. While Caucasian faces are known to be more chiseled than Asian faces because of genetics, male faces are also found to be more chiseled than female faces because of testosterone (Kurosumi et al., 2022). For example, men’s brow bones protrude further than women’s, and their eyebrows are typically straighter. If the prominent facial features of a chiseled face made emotional expressions easier to be perceived and recognized, we should have also observed the AFERSs better at recognizing emotions for male than female faces. However, it was not the case—both AFERSs classified facial expressions equally accurately for male and female faces, regardless of the datasets used (Table 3). Therefore, the discrepancy in the recognition accuracy across races is unlikely driven by the differences in the three-dimensional facial structure across races.

The discrepancy in the recognition accuracy across races can be seen as the machine version of the well-known cross-race effect in emotion recognition—people are quicker and better at recognizing and interpreting emotional facial expressions in members of their own race than in members of other races (Elfenbein and Ambady, 2002). In humans, such a cross-race effect is not innate but developed through learning (Chien et al., 2016). Specifically, through development, one will learn to pay attention to the statistical regularities of facial features useful for judging the facial expressions of others in daily life. However, such statistical regularities may vary across races owing to physical differences in face geometry or across ethnicities owing to cultural differences in facial expressions. During facial emotion recognition, visual attention biased toward a specific set of facial features regular in one race can then preclude processing of other facial features useful for judging people of the other races.

The cause of the cross-race effect in the AFERSs is likely identical to that of humans. Because AFERSs based on (deep) neural networks also inductively learn the statistical regularities in face images for judgments, they can also develop the cross-race effect if they have little or no exposure to the faces of other races (Dailey et al., 2010). This cross-race effect is only a special case of inductive biases in machine learning. In general, there is a cross-dataset generalizability issue in that AFERSs trained on one dataset often perform poorly on another dataset (Mayer et al., 2014; Li and Deng, 2018). Therefore, there are endeavors to make AFERSs learn dataset-invariant features to ensure their generalization capabilities (Chen et al., 2022; Verma et al., 2022). In the present study, our evaluated AFERSs were likely trained more on young Western faces because data on older or Eastern people are relatively scarce (Tu et al., 2018). As a result, the AFERSs did not learn the statistical regularities in Eastern faces sufficiently to make accurate judgments for Eastern faces, especially those of older adults.

In addition to the discrepancy in recognition accuracy across races, there is also a human-like discrepancy in recognition accuracy across emotion categories in the AFERSs. In the human literature, it has been long known that people can recognize facial expressions of happiness better than those of negative emotions (Palermo and Coltheart, 2004; Kosonogov and Titova, 2019). Because the AFERSs evaluated in the present study supervisedly learned from human annotations of facial expressions, they also showed the human tendency to be much better at recognizing happiness than negative expressions on faces. Therefore, despite the overall low accuracy of these AFERSs on non-Western faces, the measurements of happiness outputted by these systems are accurate across races and can still be utilized for cross-cultural studies of positive psychology.

It is worth noting that these AFERSs, due to their human-like behaviors, can serve as computational models of humans. In particular, the sample-dependent statistical learning in these AFERSs is analogous to experience-shaped perceptual learning in humans. Such shared developmental processes and decision tendencies between AFERSs and humans allow researchers to treat these artificial agents as human research participants for experimentation in face processing. For example, they can be used for predicting human participants’ decisions in the selection of face stimuli or for scrutinizing the mechanisms of the cross-race effect, among other biases in cognitive and emotional processing (Huang, 2019).

In conclusion, AFERSs, while providing objective and rapid assessments of facial expressions, should be used with caution. On the one hand, we recommend that users of these systems carry out a small-scale validation on a subset of the face images or videos of interest before adopting these automated assessments. Otherwise, problematic assessments of facial expressions can lead to incorrect understanding and even harmful interventions at both the individual and group levels. On the other hand, we also recommend that developers of these AFERSs include more non-Western samples in the training data to eliminate the data distribution bias toward Western faces. Otherwise, these AFERSs are just WEIRD tools suitable for studying WEIRD psychology (Henrich et al., 2010).

## Data availability statement

The datasets presented in this study can be found in online repositories. The names of the repository/repositories and accession number(s) can be found below: https://osf.io/m6kfy.

## Ethics statement

Ethical review and approval was not required for the study on human participants in accordance with the local legislation and institutional requirements. Written informed consent from the participants was not required to participate in this study in accordance with the national legislation and the institutional requirements.

## Author contributions

Y-TL contributed to conceptualization, investigation, and writing of the original draft. S-LY contributed to conceptualization, methodology, funding acquisition, supervision, and writing review and editing. T-RH contributed to conceptualization, methodology, funding acquisition, supervision, and writing original draft, review, and editing. All authors contributed to the article and approved the submitted version.
